# A critical residue in the α_1_M2–M3 linker regulating mammalian GABA_A_ receptor pore gating by diazepam

**DOI:** 10.7554/eLife.64400

**Published:** 2021-02-16

**Authors:** Joseph W Nors, Shipra Gupta, Marcel P Goldschen-Ohm

**Affiliations:** University of Texas at Austin, Department of NeuroscienceAustinUnited States; Universidad Nacional Autónoma de MéxicoMexico; National Institute of Neurological Disorders and Stroke, National Institutes of HealthUnited States

**Keywords:** benzodiazepine, diazepam, GABAA receptor, M2-M3 linker, allosteric modulator, *Xenopus*

## Abstract

Benzodiazepines (BZDs) are a class of widely prescribed psychotropic drugs that modulate activity of GABA_A_ receptors (GABA_A_Rs), neurotransmitter-gated ion channels critical for synaptic transmission. However, the physical basis of this modulation is poorly understood. We explore the role of an important gating domain, the α_1_M2–M3 linker, in linkage between the BZD site and pore gate. To probe energetics of this coupling without complication from bound agonist, we use a gain of function mutant (α_1_L9'Tβ_2_γ_2L_) directly activated by BZDs. We identify a specific residue whose mutation (α_1_V279A) more than doubles the energetic contribution of the BZD positive modulator diazepam (DZ) to pore opening and also enhances DZ potentiation of GABA-evoked currents in a wild-type background. In contrast, other linker mutations have little effect on DZ efficiency, but generally impair unliganded pore opening. Our observations reveal an important residue regulating BZD-pore linkage, thereby shedding new light on the molecular mechanism of these drugs.

## Introduction

Benzodiazepines (BZDs) (e.g. Valium, Xanax) are one of the most widely prescribed psychotropic drugs today. An estimated nearly 100 million adults in the United States are prescribed a BZD annually (Agarwal and Landon, 2019; Bachhuber et al., 2016; Olfson et al., 2015). Their anxiolytic and sedative properties are used as therapies for conditions including anxiety, panic, insomnia, seizures, muscle spasms, pain, and alcohol withdrawal (Möhler et al., 2002). Although largely effective, BZDs have undesirable effects, including tolerance, addiction, dependence, and withdrawal symptoms, and are often co-abused with alcohol and opioids (Fluyau et al., 2018; Schmitz, 2016; Jones and McAninch, 2015; Jones et al., 2010). Novel therapies with reduced risks are imperative for safer long-term treatment options.

**Figure 1.**
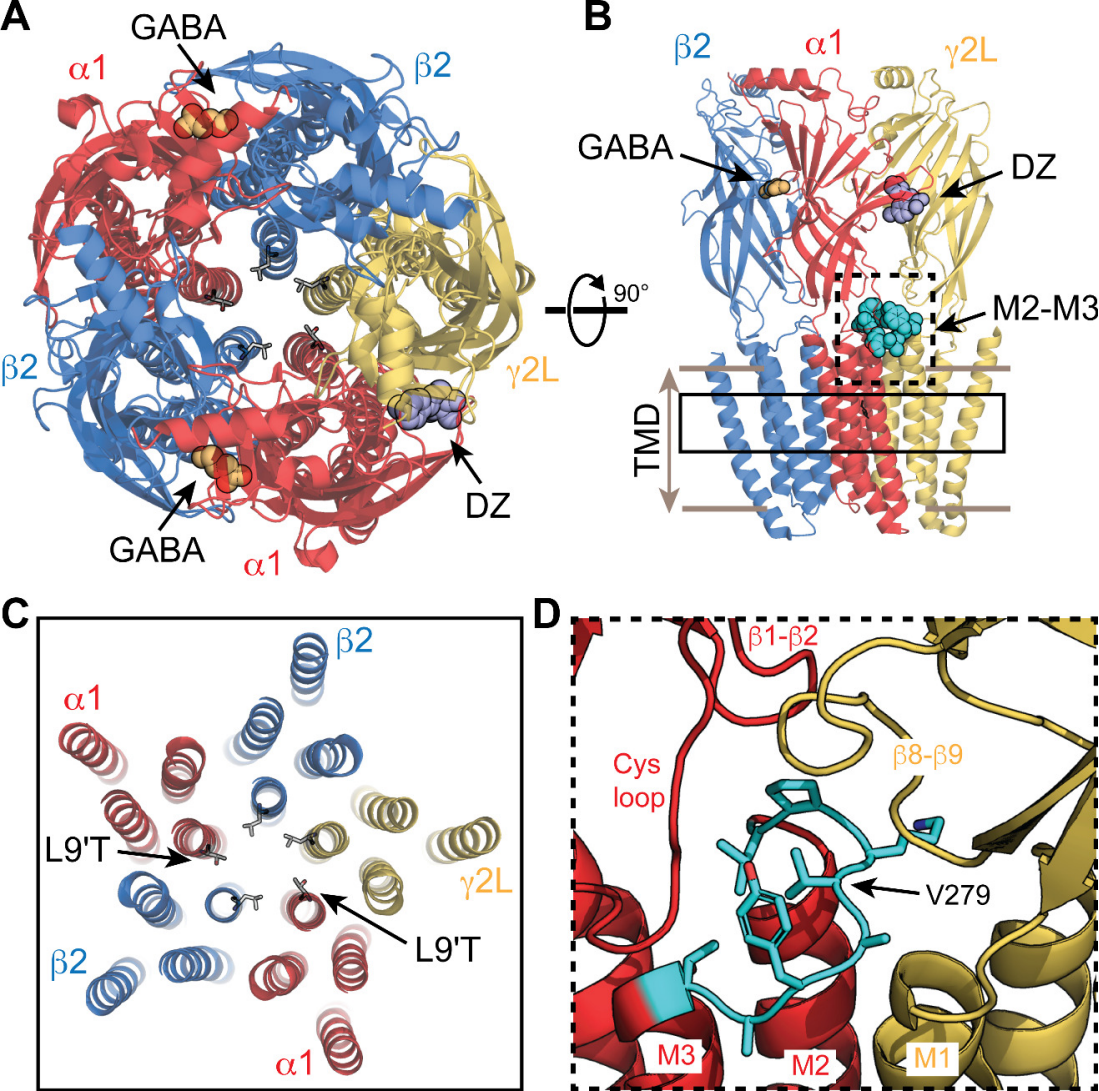
Visual representation of an α_1_β_2_γ_2_ GABA_A_ receptor from cryo-EM map PDB 6X3X. (**A,B**) View from the extracellular space (**A**) and parallel to the membrane plane (**B**). Bound GABA and DZ are shown as gold and lavender spheres, respectively. The 9' pore residue from each subunit is shown in stick representation, all leucine except for the mutation α_1_L9'T, which was generated in PyMol as a visualization aid. One of the two α_1_M2–M3 linkers is shown as cyan spheres in (**B**). (**C**) Same view as in (**A**) for a slice through the transmembrane domains indicated by the solid box in (**B**). (**D**) Detail for the dashed box in (**B**). The α_1_M2–M3 linker (L276-T283; rat numbering) is colored cyan with side chains shown in stick representation.

The therapeutic effects of BZDs are conferred upon binding to and modulating the activity of GABA_A_Rs, which are the primary inhibitory neurotransmitter-gated ion channels in the central nervous system (Smart and Stephenson, 2019). GABA_A_Rs are part of the Cys-loop superfamily of pentameric ligand-gated ion channels (pLGICs) including homologous glycine (Gly), nicotinic acetylcholine (nACh), serotonin type 3 (5-HT_3_), and zinc-activated receptors as well as prokaryotic homologs (Pless and Sivilotti, 2018; Nemecz et al., 2016; Tasneem et al., 2004; Connolly and Wafford, 2004). Each pentameric GABA_A_R is comprised of subtype-specific combinations of five homologous but nonidentical subunits (α_1-6_, β_1-3_, γ_1-3_, δ, ε, π, θ, ρ_1-3_) that together form a central chloride conducting pore (Figure 1; Olsen and Sieghart, 2008). The most prominent subtype at synapses is comprised of α_1_, β_n_, and γ_2_ subunits (Sieghart and Sperk, 2002). GABA_A_Rs provide a critical balance with excitatory signaling for normal neural function, and not surprisingly, their dysfunction is related to disorders such as epilepsy, autism spectrum disorder, intellectual disability, schizophrenia, and neurodevelopmental disorders such as fragile X syndrome (Hernandez and Macdonald, 2019; Gao et al., 2018; Mahdavi et al., 2018; Braat and Kooy, 2015; Vien et al., 2015; Rudolph and Möhler, 2014; Limon et al., 2012; Ramamoorthi and Lin, 2011; Solís-Añez et al., 2007). Although pharmacological manipulation of GABA_A_Rs is a powerful approach to tuning neural signaling, the rational design of novel therapies is challenged by a lack of understanding of the molecular mechanism by which drugs such as BZDs modulate channel behavior.

**Figure 1—figure supplement 1.**
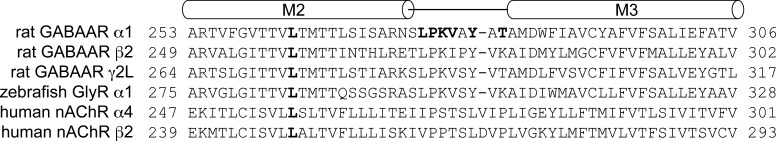
Sequence alignment of M2–M3 linker regions for subunits from several members of the pLGIC superfamily. Position of M2 and M3 helices are indicated above the sequences. The pore gate L9' residue and mutated M2–M3 linker residues are shown in bold.

In conjunction with structural models of mostly homomeric pLGICs, recent cryo-EM models of heteromeric GABA_A_Rs with bound neurotransmitter and BZD provide an important conceptual aid to understand the mechanism of action of these drugs (Kim et al., 2020; Masiulis et al., 2019; Laverty et al., 2019; Phulera et al., 2018; Zhu et al., 2018). Consistent with earlier functional studies (Sigel and Ernst, 2018; Sigel and Lüscher, 2011; Sigel, 2002; Wagner and Czajkowski, 2001; Kucken et al., 2000; McKernan et al., 1998; Pritchett et al., 1989; Gibbs et al., 1985), they show that BZDs are bound at a specific recognition site in the extracellular domain between α and γ subunits (Figure 1). However, these structures have yet to clarify the mechanism by which BZDs modulate channel activity.

Kinetic models of interacting domains and thermodynamic linkage analysis can be used to estimate the energy of interaction between binding sites and the pore gate from bulk measures of ensemble average activity (Goldschen-Ohm et al., 2014; Chowdhury and Chanda, 2013; Wyman and Gill, 1990). Coupled with mutagenesis or other perturbations, these interactions can be probed to elucidate their physical basis. However, these approaches are challenged for BZDs because they do not evoke robust channel opening by themselves, but instead modulate responses to an agonist such as GABA. Thus, typical experimental measures reflect channels with both agonist and BZD bound. This makes it difficult to dissect whether the drug has altered either agonist binding or channel gating as the two processes are intimately coupled (Colquhoun, 1998). This challenge has contributed to differing conclusions postulating that BZDs alter either agonist binding (Goldschen-Ohm et al., 2010; Perrais and Ropert, 1999; Mellor and Randall, 1997; Rogers et al., 1994; Vicini et al., 1987), pore gating (Li et al., 2013; Downing et al., 2005; Campo-Soria et al., 2006; Rüsch and Forman, 2005), or an intermediate preactivation step (Goldschen-Ohm et al., 2014; Gielen et al., 2012). The ability of BZDs to potentiate current responses to saturating concentrations of partial agonists, and also to directly gate gain of function mutants, implies that the drug’s effect is not due to changes in binding alone. A combination of effects on both binding and gating as predicted by changes in intermediate gating steps energetically coupled with both binding sites and the pore gate is plausible (Goldschen-Ohm et al., 2014). However, the molecular identity of any such intermediate states remains unclear.

Here we examined linkage between the BZD site and pore gate in isolation from any effect of the drug on agonist binding using a spontaneously active gain of function mutant (α_1_L9'Tβ_2_γ_2L_) that is directly gated by BZDs alone (Scheller and Forman, 2002; Chang and Weiss, 1999). In the α_1_L9'T background, we serially mutated each residue in the α_1_M2–M3 linker to alanine and assessed the effect on modulation of the channel pore by the BZD positive modulator DZ (Valium) in the absence of agonist. The M2–M3 linker is a loop following the pore lining M2 helix that together with several other important interfacial loops defines the region connecting extracellular ligand binding and transmembrane domains known to be crucially involved in the gating process of pLGICs (Figure 1). Structural models show that agonist binding is associated with an outward displacement of the M2–M3 linker from the central pore axis (Nemecz et al., 2016). Mutations in the linker generally impair gating by agonists (Kash et al., 2003; O'Shea and Harrison, 2000), and some are associated with genetic diseases such as epilepsy in GABA_A_Rs (Hales et al., 2006; Hernandez and Macdonald, 2019) or hyperekplexia in GlyRs (Bode and Lynch, 2014). However, the role of the M2–M3 linker in drug modulation by BZDs is less understood.

We show that alanine substitutions throughout the α_1_M2–M3 linker generally impair unliganded pore gating, whereas they have little effect on the efficiency by which chemical energy from DZ binding is transmitted to the pore gate. The notable exception is α_1_V279A, which more than doubles DZ’s energetic contribution to pore opening, whereas larger side chains at this site do not. In a wild-type background, α_1_V279A enhances DZ potentiation of currents evoked by even saturating GABA, consistent with a direct effect on the pore closed/open equilibrium. Our observations identify an important residue in the α_1_M2–M3 linker regulating the efficiency of BZD modulation in GABA_A_Rs.

## Results

### Alanine substitutions in the α_1_M2–M3 linker generally impair unliganded gating

**Figure 2.**
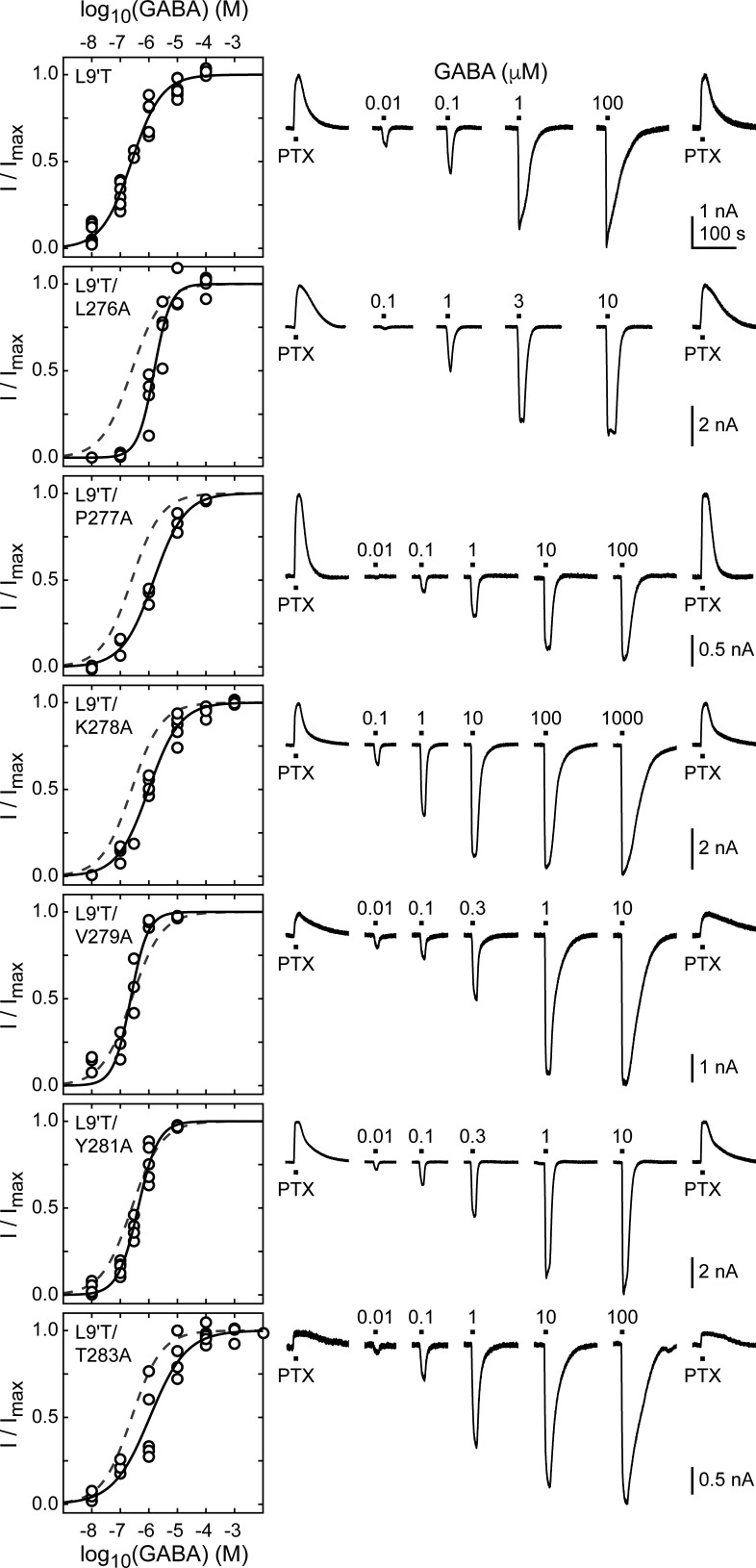
Spontaneous PTX-sensitive and GABA-evoked currents for α_1_M2–M3 linker alanine substitutions in the gain of function α_1_L9'Tβ_2_γ_2L_ background. (Left) Summary of normalized GABA concentration–response relationships for GABA-evoked currents with the zero current baseline set to the level of spontaneous activity. Solid line is a fit of the pooled data across oocytes to Equation 1, and the dashed line is the fit for the L9'T background. Fit parameters are EC_50_, hill slope (# oocytes): L9'T = 0.25 μM, 0.83 (7); L9'T/L276A = 1.53 μM, 1.51 (4); L9'T/P277A = 1.48 μM, 0.80 (3); L9'T/K278A = 0.99 μM, 0.79 (5); L9'T/V279A = 0.23 μM, 1.44 (4); L9'T/Y281A = 0.42 μM, 1.23 (5); L9'T/T283A = 1.08 μM, 0.67 (5). Parameters for fits to individual oocytes are summarized in Figure 2—figure supplement 2. (Right) Example currents in response to 10 second pulses of the pore blocker PTX (1 mM) or GABA (concentration in micromolar indicated above each pulse). Responses to GABA were bookended by application of PTX to assess the amount of spontaneous current and to normalize any drift or rundown during the experiment (see Figure 2—figure supplement 1).

We use the gain of function mutation α_1_L9'T that confers spontaneous channel gating in the absence of agonist that can be further modulated by BZDs (Scheller and Forman, 2002; Chang and Weiss, 1999). The primary purpose for this mutation is to enable macroscopic current responses to report on pore gating by a BZD in the absence of agonist. Initially, we examine unliganded and GABA-evoked gating and in later sections turn to BZDs. In the α_1_L9'Tβ_2_γ_2L_ (α_1_L9'T or L9'T) gain of function background, we assessed the effects of alanine substitutions in the α_1_M2–M3 linker on unliganded and GABA-evoked channel activity. We restricted the scan primarily to the flexible loop between helical regions associated with M2 and M3 from α_1_L276-T283 (Figure 1; Figure 1—figure supplement 1). Two of these positions, α_1_A280 and α_1_A282, are natively alanine and were not mutated. Briefly, *Xenopus laevis* oocytes were co-injected with mRNA for α, β, and γ subunits in a 1:1:10 ratio, and current responses to microfluidic application of ligands were recorded in two-electrode voltage clamp. Each recording consisted of a series of 10 second pulses of GABA at various concentrations bookended by 10 second pulses of 1 mM picrotoxin (PTX) (Figure 2). Current block by the pore blocker PTX was used to assess the amount of spontaneous activity and to correct for drift or rundown over the course of the experiment (see Figure 2—figure supplement 1). In comparison, wild-type receptors have little to no PTX-sensitive spontaneous activity (Figure 2—figure supplement 3). Normalized concentration–response relationships for the additional GABA-evoked current above the spontaneous current baseline in the L9'T background are shown in Figure 2. As compared to α_1_L9'T, all of the alanine mutations exhibited an increase in the EC_50_ and/or steepness of the GABA concentration–response relationship (Figure 2; Figure 2—figure supplement 2). The increased EC_50_ is generally consistent with previous reports of α_1_M2–M3 linker mutations in a wild-type background (O'Shea and Harrison, 2000) and implies that side chain interactions in this region are important for channel gating by agonists. The steepness of the concentration–response relationship for α_1_L9'T was fairly shallow, with a hill slope slightly below one. This could reflect an increased efficiency of mono-liganded gating in the gain of function background. In such a case, the steeper hill slopes conferred by α_1_L276A, α_1_V279A, and α_1_Y281A may be due to a reduction in the efficiency of mono- vs di-liganded gating, although this was not verified.

**Figure 2—figure supplement 1.**
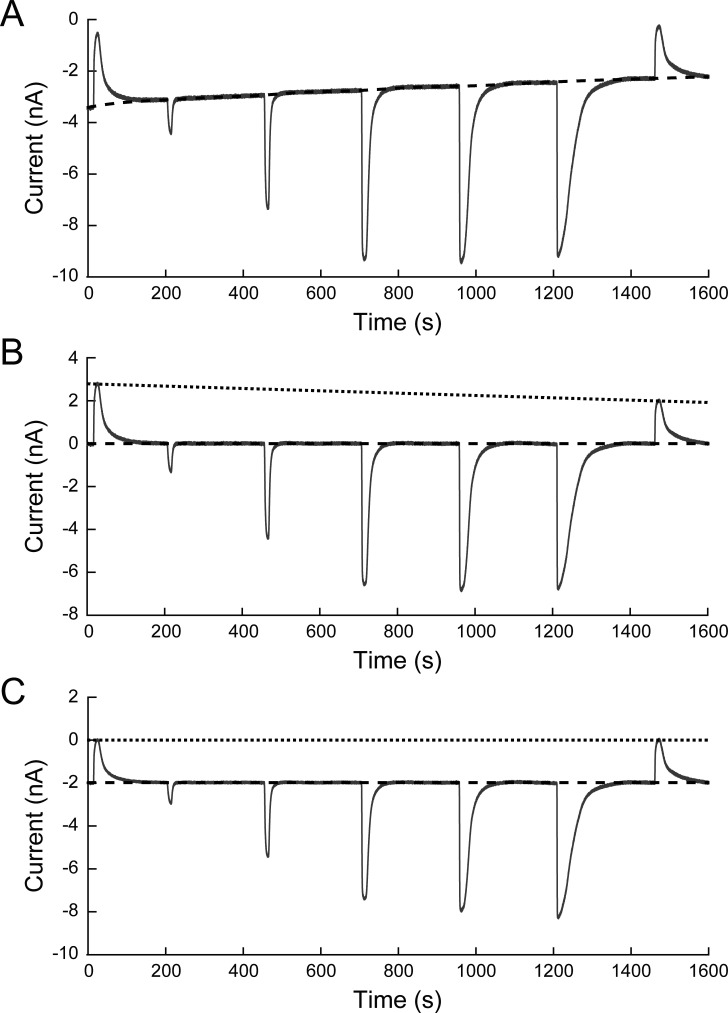
Correction for baseline drift and current rundown. (**A**) Raw two-electrode voltage clamp current recording from α_1_L9'T/K278Aβ_2_γ_2L_ receptors expressed in a *Xenopus laevis* oocyte. Responses to 10 second pulses of increasing concentrations of GABA (0.1, 1, 10, 100, and 1000 μM) were bookended by 10 second pulses of 1 mM PTX as shown in Figure 2. (**B**) Baseline drift was corrected by subtracting a spline fit to selected segments of baseline (dashed line in **A**). (**C**) Current rundown was corrected by linearly scaling the current record, so that the bookending PTX responses were of equal amplitude (dotted line in **B**). The resulting current trace was shifted, so that zero current coincides with the peak response to block by PTX.

**Figure 2—figure supplement 2.**
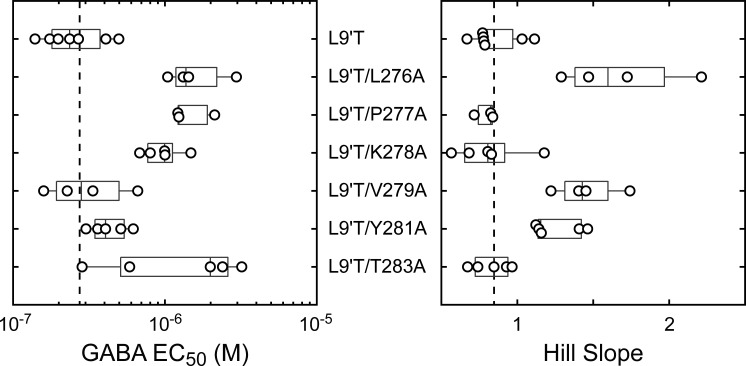
Summary of GABA EC_50_ and hill slope from fitting Equation 1 to GABA concentration–response relationships as shown in Figure 2 for individual oocytes. Gray box plots indicate the median and 25th and 75th percentiles. The vertical dashed line is the mean for L9'T.

**Figure 2—figure supplement 3.**
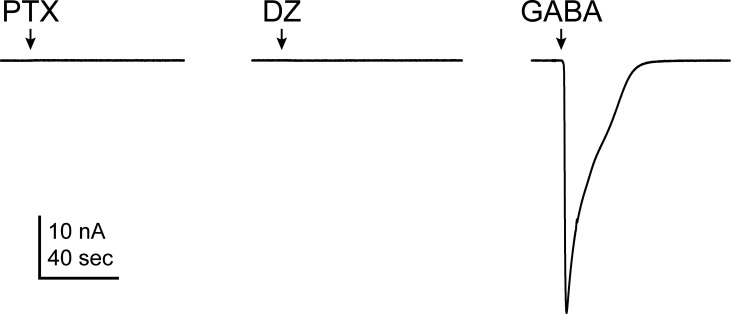
Responses of wild-type α_1_β_2_γ_2L_ receptors to 10 second pulses of either 1 mM PTX, 3 μM DZ, or 3 mM GABA.

**Figure 3.**
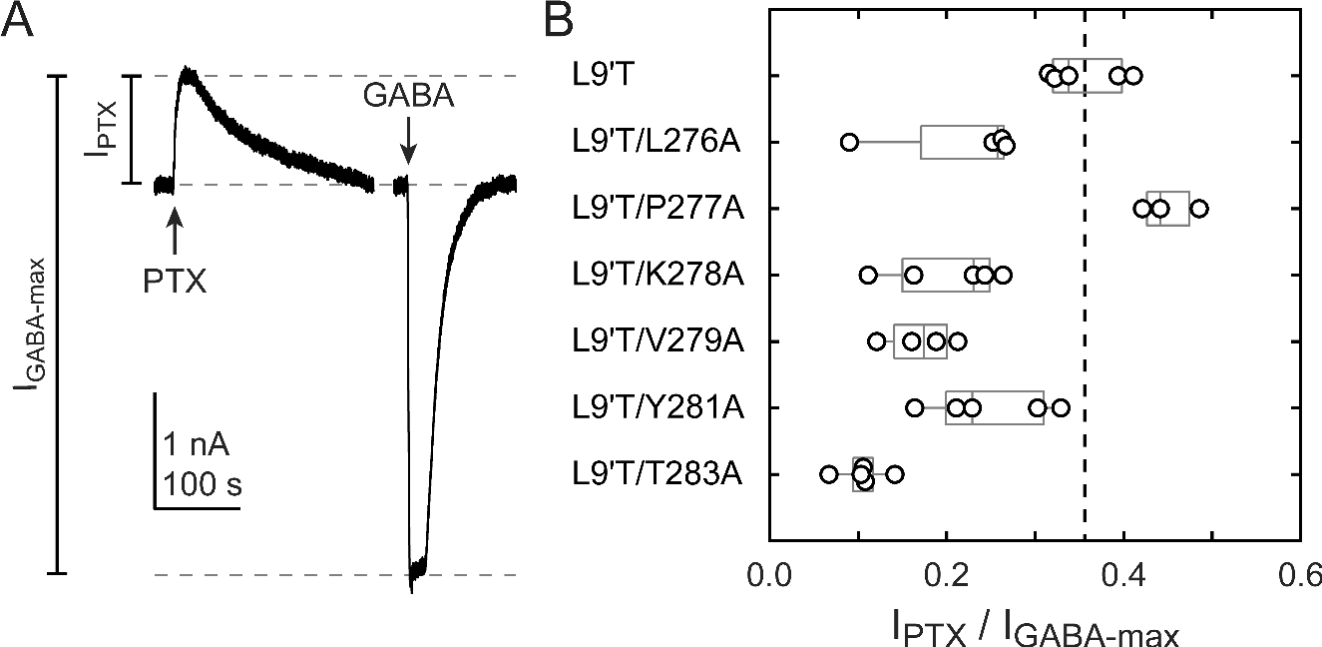
Ratio of PTX-sensitive to maximal GABA-evoked current amplitude. (**A**) Example currents from α_1_L9'T/V279Aβ_2_γ_2L_ receptors elicited by 10 second pulses of either 1 mM PTX or 10 μM GABA. (**B**) Summary of the ratio of PTX-sensitive to maximal GABA-evoked current amplitude for individual oocytes. Gray box plots indicate the median and 25th and 75th percentiles. The vertical dashed line is the mean for L9'T. These ratios were estimated as being approximately proportional to the unliganded open probability by a factor that is the open probability in saturating GABA.

The level of unliganded channel activity was determined from the ratio of the spontaneous current amplitude as assessed by block with PTX (I_PTX_) to the maximal current amplitude evoked with saturating GABA (I_GABA-max_) (Figure 3A). The unliganded open probability was estimated as the product of the ratio I_PTX_/I_GABA-max_ and the open probability in saturating GABA (P_o-GABA-max_). Under the assumption that P_o-GABA-max_ is similar for all constructs, all α_1_M2–M3 linker mutations except for α_1_P277A reduced the unliganded open probability by approximately two-fold (Figure 3B, Table 1). P_o-GABA-max_ is ~0.85 in wild-type channels (Keramidas and Harrison, 2010), and likely to be closer to 1.0 in a gain of function background such as α_1_L9'T that is further known to desensitize much more slowly than wild-type (Scheller and Forman, 2002). This assumption was verified for single L9'T/V279A receptors (Figure 7—figure supplement 1). Thus, any reduction in P_o-GABA-max_ for the linker mutations should only further reduce their estimated unliganded open probability. In summary, these data show that alanine mutations in the α_1_M2–M3 linker generally impair channel gating by increasing GABA EC_50_ and/or reducing unliganded pore opening. This suggests that α_1_M2–M3 linker side chain interactions play an important role in the closed vs. open pore equilibrium even in the absence of agonist.

### All but one alanine substitution in the α_1_M2–M3 linker have little effect on activation by DZ relative to unliganded activity

**Figure 4.**
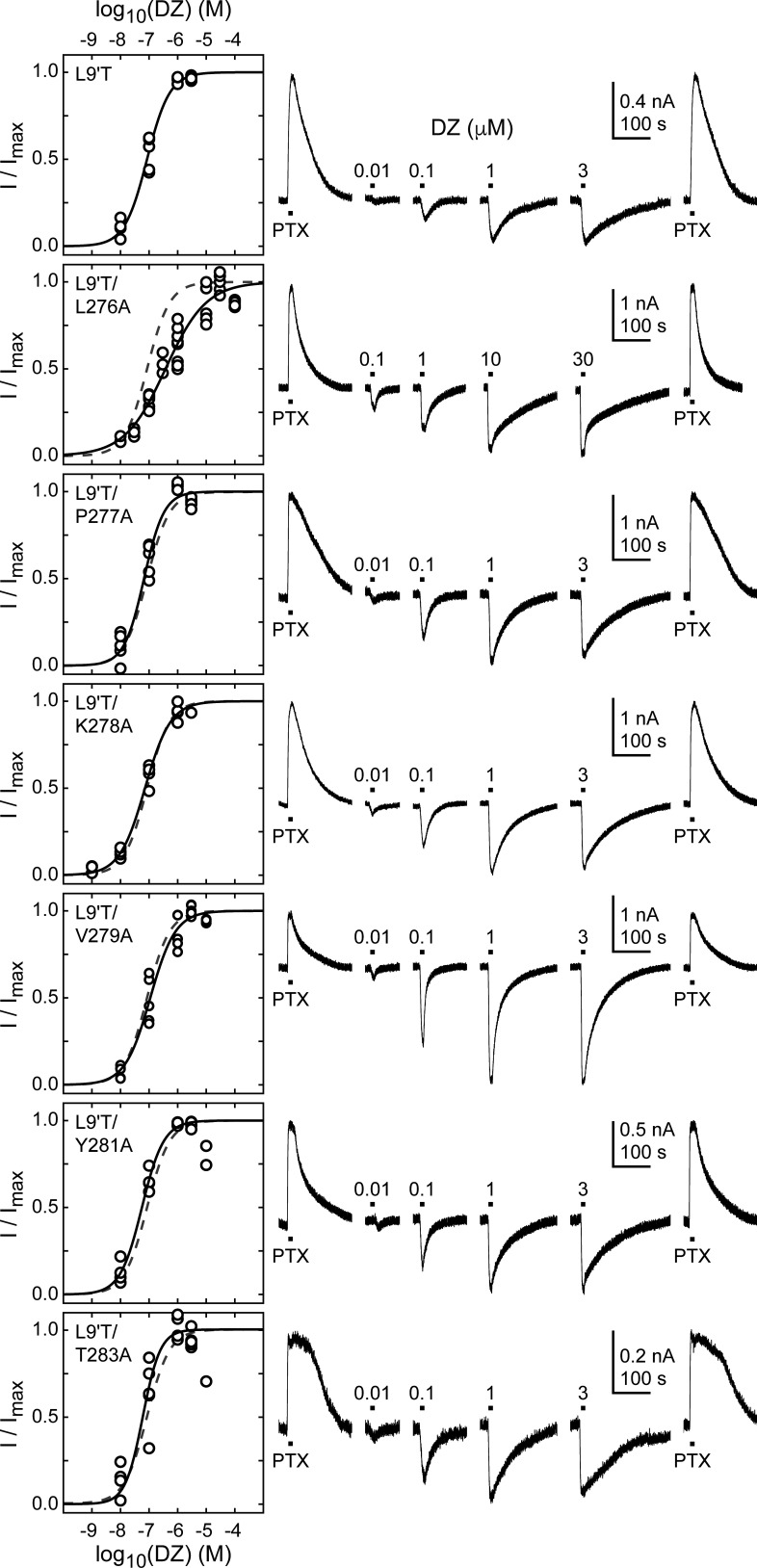
Spontaneous PTX-sensitive and DZ-evoked currents for α_1_M2–M3 linker alanine substitutions in the gain of function α_1_L9'Tβ_2_γ_2L_ background. (Left) Summary of normalized DZ concentration–response relationships for DZ-evoked currents with the zero current baseline set to the level of spontaneous activity. Solid line is a fit of the pooled data across oocytes to Equation 1, and the dashed line is the fit for the L9'T background. Reduced peak responses to 10 μM DZ for L9'T/Y281A and L9'T/T283A were omitted from the fits. Fit parameters are EC_50_, hill slope (# oocytes): L9'T = 85 nM, 1.10 (5); L9'T/L276A = 380 nM, 0.61 (8); L9'T/P277A = 67 nM, 1.24 (5); L9'T/K278A = 72 nM, 0.97 (4); L9'T/V279A = 110 nM, 0.98 (5); L9'T/Y281A = 56 nM, 1.14 (4); L9'T/T283A = 60 nM, 1.42 (6). Parameters for fits to individual oocytes are summarized in Figure 4—figure supplement 1. (Right) Example currents in response to 10 second pulses of the pore blocker PTX (1 mM) or DZ (concentration in micromolar indicated above each pulse). Responses to DZ were bookended by application of PTX to assess the amount of spontaneous current and to normalize any drift or rundown during the experiment (see Figure 2—figure supplement 1).

BZD positive modulators alone evoke channel opening with extremely low probability (Campo-Soria et al., 2006), thus necessitating coapplication with an agonist to obtain robust currents from wild-type receptors (Germann et al., 2019). However, dissecting the effects of BZDs on either agonist binding or channel gating is severely challenged in the presence of an agonist because the two processes are intimately coupled (Colquhoun, 1998). To isolate effects on gating apart from any effects on agonist binding, we examined the effect of alanine substitutions in the α_1_M2–M3 linker on DZ-evoked currents in the background of the gain of function mutation α_1_L9'T in the absence of agonist. Concentration–response relationships for currents evoked by 10 second pulses of DZ were bookended by 10 second pulses of 1 mM PTX to assess spontaneous activity (Figure 4). Drift or rundown was corrected as described for GABA-evoked currents (Figure 2—figure supplement 1). Normalized concentration–response relationships for the additional DZ-elicited current above the spontaneous current baseline were fit to Equation 1. Alanine substitutions in the α_1_M2–M3 linker have no obvious effect on DZ concentration–response relationships with the exception of α_1_L276A that confers a right shift and reduction in steepness (Figure 4; Figure 4—figure supplement 1). Reports for the EC_50_ of DZ potentiation in wild-type receptors are similar to the DZ EC_50_ for α_1_L9'T receptors reported here and in previous studies (Li et al., 2013; Campo-Soria et al., 2006; Rüsch and Forman, 2005; Walters et al., 2000).

**Figure 4—figure supplement 1.**
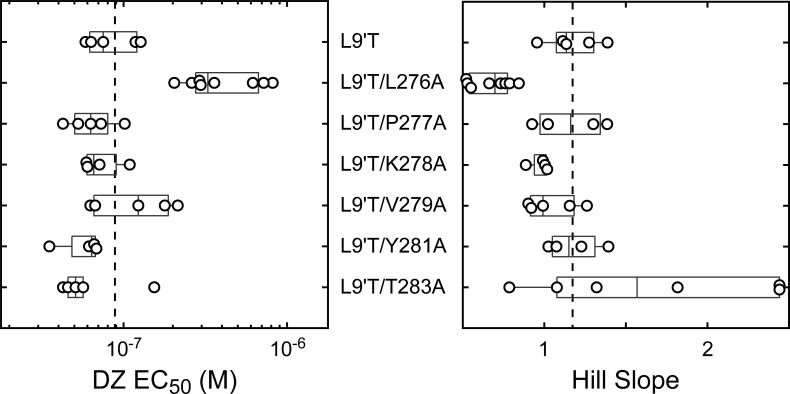
Summary of DZ EC_50_ and hill slope from fitting Equation 1 to DZ concentration–response relationships as shown in Figure 4 for individual oocytes. Gray box plots indicate the median and 25th and 75th percentiles. The vertical dashed line is the mean for L9'T.

**Figure 5.**
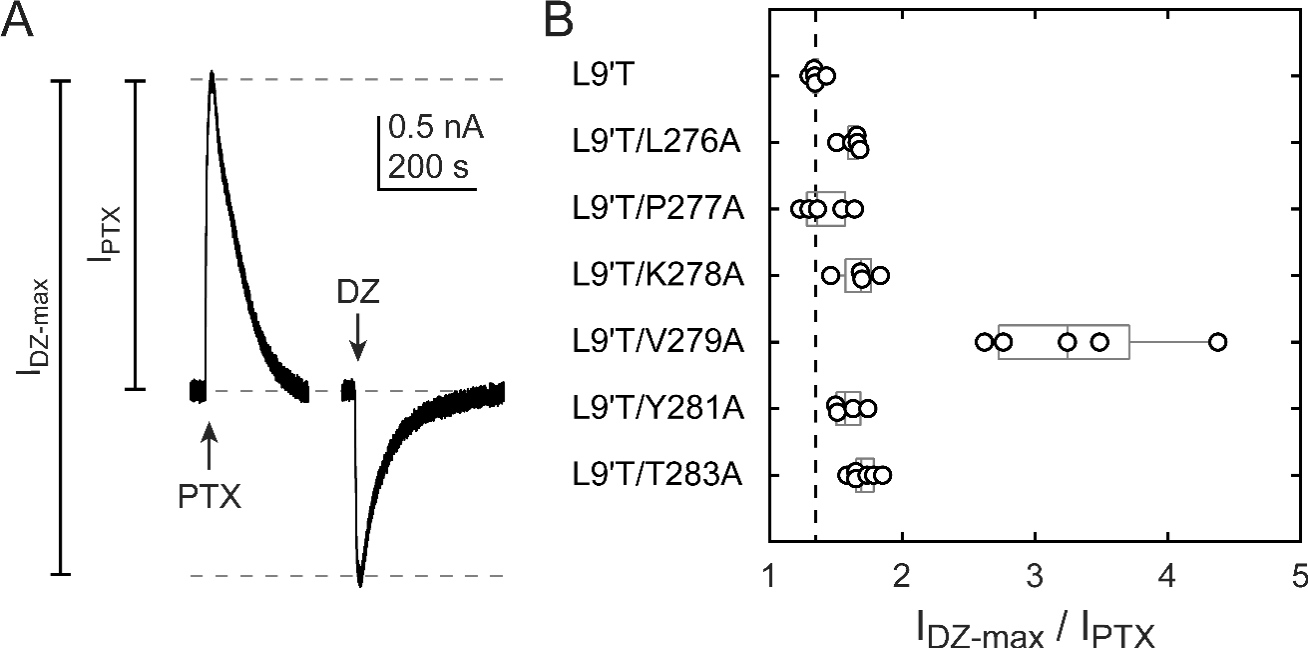
Ratio of maximal DZ-evoked to PTX-sensitive current amplitude. (**A**) Example currents from α_1_L9'T/K278Aβ_2_γ_2L_ receptors elicited by 10 second pulses of either 1 mM PTX or 1 μM DZ. (**B**) Summary of the ratio of maximal DZ-evoked to PTX-sensitive current amplitude for individual oocytes. Gray box plots indicate the median and 25th and 75th percentiles. The vertical dashed line is the mean for L9'T. These ratios were used as estimates for the approximate fold-change in open probability upon DZ binding.

The ratio of the maximal DZ-evoked current amplitude (I_DZ-max_) to PTX-sensitive current amplitude (I_PTX_) is a measure for how well DZ activates the channel relative to its spontaneous unliganded activity. This ratio is largely independent of alanine substitution in the α_1_M2–M3 linker with the notable exception of α_1_V279A (Figure 5, Table 1). Thus, apart from α_1_V279A, DZ-evoked currents are predictably proportional to the amount of unliganded activity. This suggests that α_1_V279 has a unique role in DZ modulation, whereas side chains of other linker residues are involved little or not at all.

### Dependence of DZ gating on charge and volume at α_1_V279

**Figure 6.**
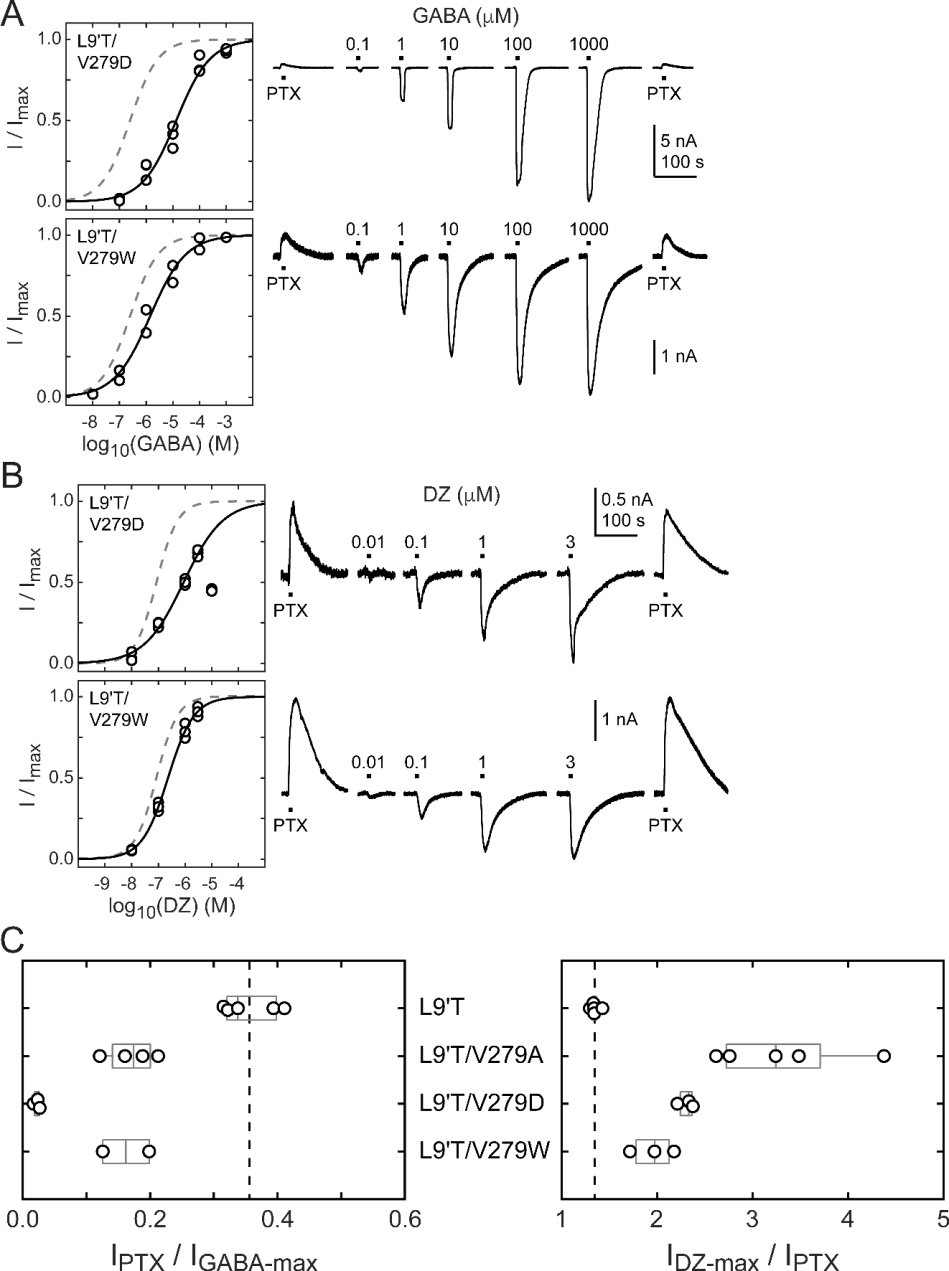
Spontaneous PTX-sensitive and GABA- or DZ-evoked currents for α_1_V279D and α_1_V279W in the gain of function α_1_L9'Tβ_2_γ_2L_ background. (**A**) Summary of normalized GABA concentration–response relationships for GABA-evoked currents with the zero current baseline set to the level of spontaneous activity (left). Solid line is a fit of the combined data across oocytes to Equation 1, and the dashed line is the fit for the L9'T background. Fit parameters are EC_50_, hill slope (# oocytes): L9'T/V279D = 13 μM, 0.68 (3); L9'T/V279W = 1.4 μM, 0.65 (2). Example currents in response to 10 second pulses of the pore blocker PTX (1 mM) or GABA (concentration in micromolar indicated above each pulse) (right). (**B**) Same as in (**A**) except for DZ-evoked currents. Responses from L9'T/V279D to 10 μM DZ were excluded from the fit. Fit parameters are EC_50_, hill slope (# oocytes): L9'T/V279D = 0.88 μM, 0.58 (3); L9'T/V279W = 0.23 μM, 0.90 (3). (**C**) Summary of the ratios of PTX-sensitive and either maximal GABA- or DZ-evoked current amplitudes for individual oocytes. Gray box plots indicate the median and 25th and 75th percentiles. The vertical dashed line is the mean for L9'T. These ratios were used as estimates for the approximate fold-change in open probability upon DZ binding.

The M2–M3 linker has both high sequence and structural similarity in pLGICs. The valine at position 279 in the GABA_A_R α_1_ subunit is located near the center of the linker and is nearly always an aliphatic residue in different GABA_A_R subunits as well as in subunits of other pLGICs, with the exception of AChR, where it is a threonine. Even where sequences differ, structural models of the M2–M3 linker for multiple pLGICs are highly similar. To explore the side chain properties relevant to DZ gating, we examined the effect of introducing a charged aspartate or bulky tryptophan at position 279 in the α_1_L9'T background. We recorded unliganded PTX-sensitive and GABA- or DZ-evoked currents as described above and compared their relative current amplitudes and concentration–response relationships (Figure 6). The negative charge introduced by α_1_V279D severely inhibits unliganded gating, exhibiting very little PTX-sensitive current in relation to robust currents evoked with saturating GABA. In contrast, addition of the bulky side chain α_1_V279W only slightly impairs unliganded gating to a similar degree as substitution with alanine. These data suggest that side chain volume at this position is less critical for unliganded gating, whereas introduction of a negative charge nearly abolishes spontaneous activity despite the α_1_L9'T pore mutation. Consistent with impaired pore gating, α_1_V279D increases the GABA EC_50_ by ~100-fold.

The ratio of DZ-evoked to PTX-sensitive current amplitude is only slightly increased by α_1_V279W, whereas it is further increased by α_1_V279D, albeit not to the extent of α_1_V279A (Figure 6C). However, we were unable to reach saturation for DZ-evoked currents from α_1_V279D due to a reduction in peak current amplitude at higher DZ concentrations consistent with occupation of a secondary lower affinity inhibitory site (Figure 6B). Thus, our observations for α_1_V279D reflect a lower limit on channel activity evoked by DZ binding. Even if α_1_V279D has an appreciable effect on the ratio of DZ-evoked to unliganded current, the overall effect of this mutation on channel function is likely to be dominated by its inhibition of pore gating. In contrast, α_1_V279W has similar effects to alanine substitutions at other linker residues. These data suggest that DZ gating is specifically enhanced by a reduction of side chain volume at position 279.

### The mutation α_1_V279A increases DZ’s energetic contribution to pore gating

**Figure 7.**
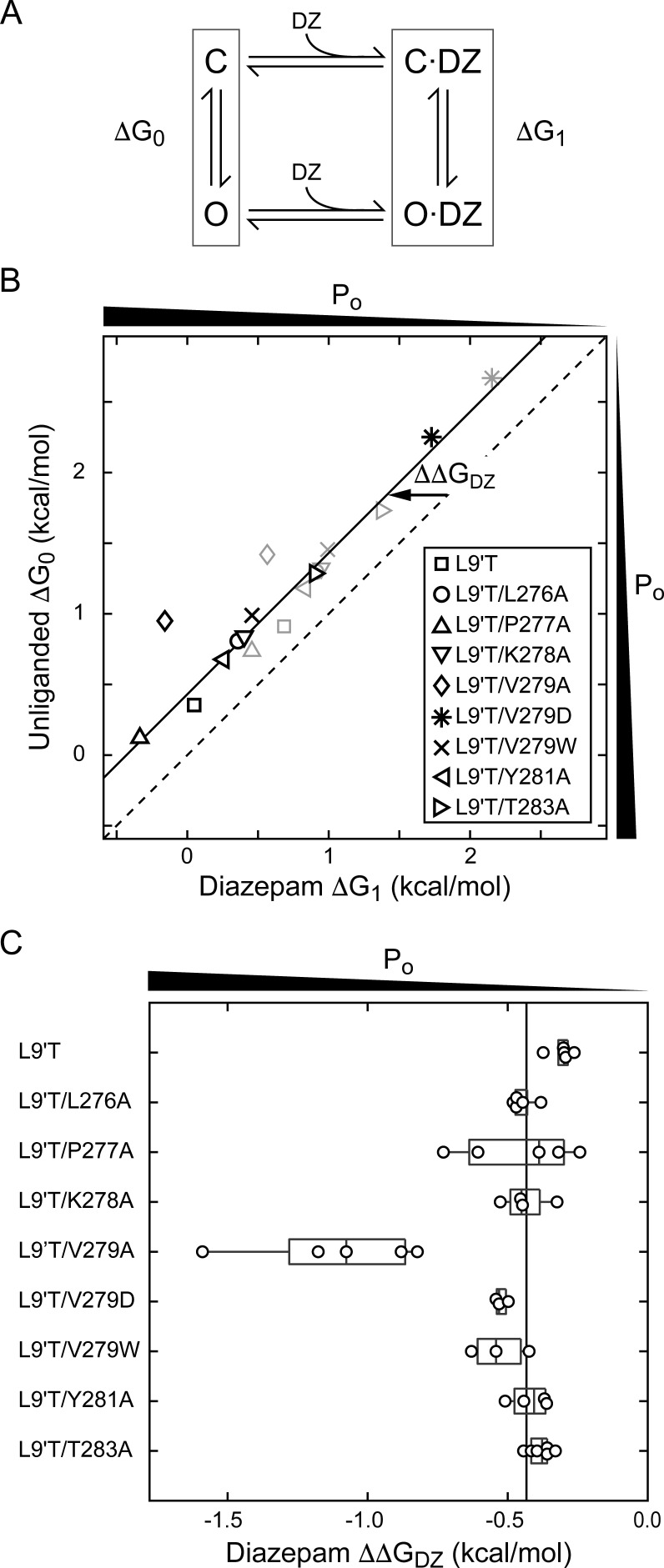
A critical residue in the α_1_M2–M3 linker regulating DZ’s energetic contribution to pore gating. (**A**) A simple model approximating channel gating between closed (C) and open (O) pore states in both unliganded and DZ-bound conditions. (**B**) Relationship between gating free energy in unliganded (ΔG_0_) and DZ-bound (ΔG_1_) conditions for α_1_L9'Tβ_2_γ_2L_ receptors and α_1_M2–M3 linker mutations assuming P_o–GABA–max _= 1 (bold symbols) or P_o–GABA–max _= 0.5 (light gray symbols) (Equations 2–3). ΔG values are the change in energy from closed to open states, such that negative values favor opening. To illustrate this, we indicate the direction of increasing open probability (P_o_) along each axis. Points are the mean across oocytes. The dashed line of symmetry reflects ΔG_0_ = ΔG_1_ where DZ would have no effect on pore gating. The solid line is a fit to ΔG_1_ = ΔG_0_ + ΔΔG_DZ_ for all of the data points except the outlier α_1_V279A given P_o-GABA-max_ = 1. The good description of the data suggests that DZ’s energetic contribution to pore gating is the same for all of the constructs on this line, estimated as ΔΔG_DZ_ = −0.4 kcal/mol. In contrast, α_1_V279A more than doubles the energy that DZ binding transmits to the pore gate. A comparison of the bold and light gray symbols shows that reducing P_o–GABA–max_ to 0.5 primarily shifts the data points along the fitted line with only minor changes in ΔΔG_DZ_, indicating that our assumption for the value of P_o–GABA–max_ is not critical for interpretation of ΔΔG_DZ_. Nonetheless, we verified that single L9'T/V279A receptors open with high probability (Figure 7—figure supplement 1). (**C**) Summary of ΔΔG_DZ_ for individual oocytes. Negative values of ΔΔG_DZ_ increase channel open probability. Gray box plots indicate the median and 25th and 75th percentiles. The vertical line is the position of linear fit in (B) corresponding to −0.4 kcal/mol.

To estimate the amount of chemical energy from DZ binding that is transmitted to the pore gate, we employed a simple model of channel gating between closed (C) and open (O) pore states in both unliganded and DZ-bound conditions (Figure 7A). The model assumes that gating can be approximated with a single closed and open states in each liganded condition. The free energy differences for pore gating in unliganded (ΔG_0_) and DZ-bound (ΔG_1_) conditions were calculated according to Equations 2–3 under the assumption that P_o-GABA-max_ ~ 1. A comparison of ΔG_0_ versus ΔG_1_ shows that DZ binding confers a uniform ΔΔG_DZ_ = ΔG_1_− ΔG_0_ = −0.4 kcal/mol to the pore gating equilibrium independent of α_1_M2–M3 linker mutation with the notable exception f α_1_V279A that more than doubles ΔΔG_DZ_ (Figure 7B,C and Table 1). Importantly, this result is largely independent of our assumption for P_o-GABA-max_ (see bold vs. light gray symbols in Figure 7B). Nonetheless, we verified that single L9'T/V279A channels in saturating GABA open with high probability to a similar maximal conductance level as wild-type and L9'T channels (Figure 7—figure supplement 1). These observations suggest that coupling between the BZD site and pore gate is relatively independent of individual α_1_M2–M3 linker side chain interactions except for α_1_V279, which natively hinders DZ gating as compared to its substitution with alanine. Strikingly, introduction of a bulky tryptophan or charged aspartate at position 279 have much smaller effects on ΔΔG_DZ_, consistent with the idea that the small side chain volume of alanine is the most relevant factor for increased DZ efficiency.

**Figure 7—figure supplement 1.**
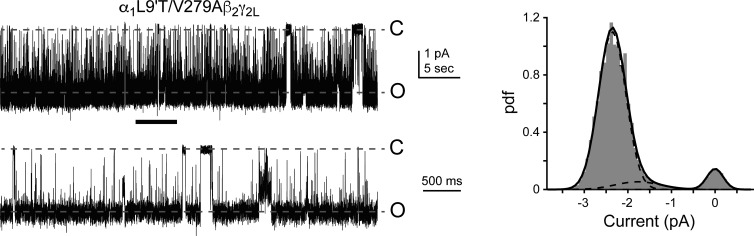
Single-channel opening for L9'T/V279A in saturating GABA. (Left) Single-channel recording for an α_1_L9'T/V279Aβ_2_γ_2L_ receptor in 100 μM GABA. Dashed lines indicate fully closed (C) and fully open (O) current levels. Bottom trace shows the portion of the top trace indicated by the black bar on an expanded timescale. At least one subconductance level was observed, although it was relatively infrequent as compared to the maximal conductance level. Trace was low-pass filtered at 1 kHz for display only. (Right) Histogram of current amplitudes from the trace on the left. Solid line is a fit to a mixture of three Gaussian components, and dashed lines show individual components. Components mean ± standard deviation (weight): −2.4 ± 0.3 (0.86), −1.8 ± 0.5 (0.07), 0.0 ± 0.2 (0.07).

### The mutation α_1_V279A enhances DZ potentiation of GABA-evoked currents in a wild-type background

**Figure 8.**
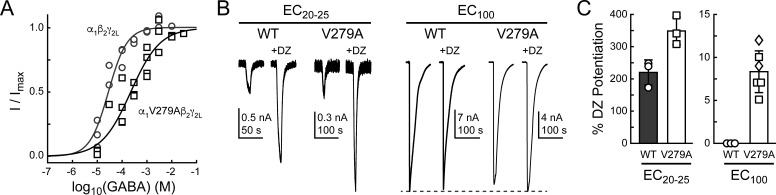
The mutation α_1_V279A enhances DZ potentiation of GABA-evoked current amplitudes in a wild-type (WT) α_1_β_2_γ_2L_ background. (**A**) Normalized GABA concentration–response relationships for WT (circles, three oocytes) and V279A (squares, four oocytes). Solid lines are fits to Equation 1 for all oocytes combined. Fit parameters are EC_50_, hill slope (# oocytes): WT = 28 μM, 1.2 (3); V279A = 222 μM, 0.7 (4). (**B**) Potentiation of current amplitudes evoked by 10 second pulses of either subsaturating EC_20-25_ (WT: 10 μM, V279A: 30 μM) or saturating EC_100_ (WT: 3 mM, V279A: 3–30 mM) GABA by 1 μM DZ. (**C**) Summary of potentiation as shown in (**B**) for individual oocytes. For V279A EC_100_, squares indicate 3 or 10 mM GABA and diamonds 30 mM GABA.

If gating in the α_1_L9'T background is relevant to neurotransmitter-driven gating in native receptors, then the mutation α_1_V279A should both impair gating by GABA and enhance DZ potentiation of GABA-evoked currents in a wild-type background. To test this, we compared GABA-evoked current responses in α_1_β_2_γ_2L_ (wild-type) and α_1_V279Aβ_2_γ_2L_ (V279A) receptors, as well as the ability of 1 μM DZ to potentiate these responses. First, GABA EC_50_ is increased by α_1_V279A, consistent with impaired unliganded gating (Figure 8A). This effect is similar, although slightly larger than previously observed in α_2_β_1_γ_2S_ receptors (O'Shea and Harrison, 2000). Second, DZ potentiates GABA-evoked peak current responses to a greater extent in V279A than in wild-type receptors, consistent with enhanced coupling between the BZD site and pore gate in the mutant (Figure 8B,C). In contrast to wild-type, DZ also potentiates V279A currents evoked with a saturating concentration of GABA. Together, these data suggest that the additional DZ modulation conferred by α_1_V279A is both independent of agonist association and additive to DZ potentiation in wild-type receptors.

**Figure 8—figure supplement 1.**
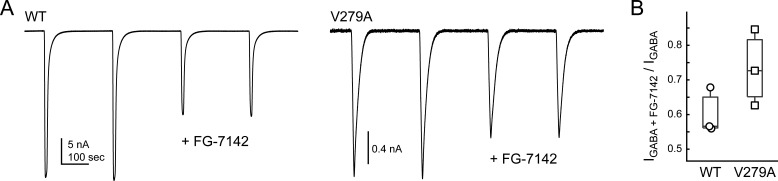
Reduction of GABA-evoked currents by the BZD negative modulator FG-7142 for α_1_β_2_γ_2L_ (WT) and α_1_V279Aβ_2_γ_2L_ (V279A) receptors. (**A**) Pair of current responses to 10 second pulses of ~EC_20-25_ GABA (WT: 10 μM, V279A: 30 μM) followed by a pair of responses to ~EC_20-25_ GABA + 1 μM FG-7142. (**B**) Summary of the ratio of peak currents as shown in (**A**) in the presence and absence of FG-7142 for individual oocytes. Gray box plots indicate the median and 25th and 75th percentiles.

In addition to the BZD positive modulator DZ, we examined the effect of the α_1_V279A mutation on the BZD negative modulator FG-7142 (Im et al., 1995). Responses to ~EC_20-25_ GABA were reduced in the presence of FG-7142 in both WT and V279A receptors, but to a slightly lesser extent in V279A (Figure 8—figure supplement 1). Distinct mechanisms for BZD positive and negative modulation could explain the observed reduction in negative modulation as compared to enhancement of positive modulation conferred by α_1_V279A. Alternatively, our observations are also consistent with the idea that α_1_V279A stabilizes an open channel configuration specifically in the BZD-bound complex and that this stabilization works in concert with positive modulators but hinders negative modulators.

### α_1_L9'T and α_1_V279A have independent and additive effects on the pore-gating equilibrium

**Figure 9.**
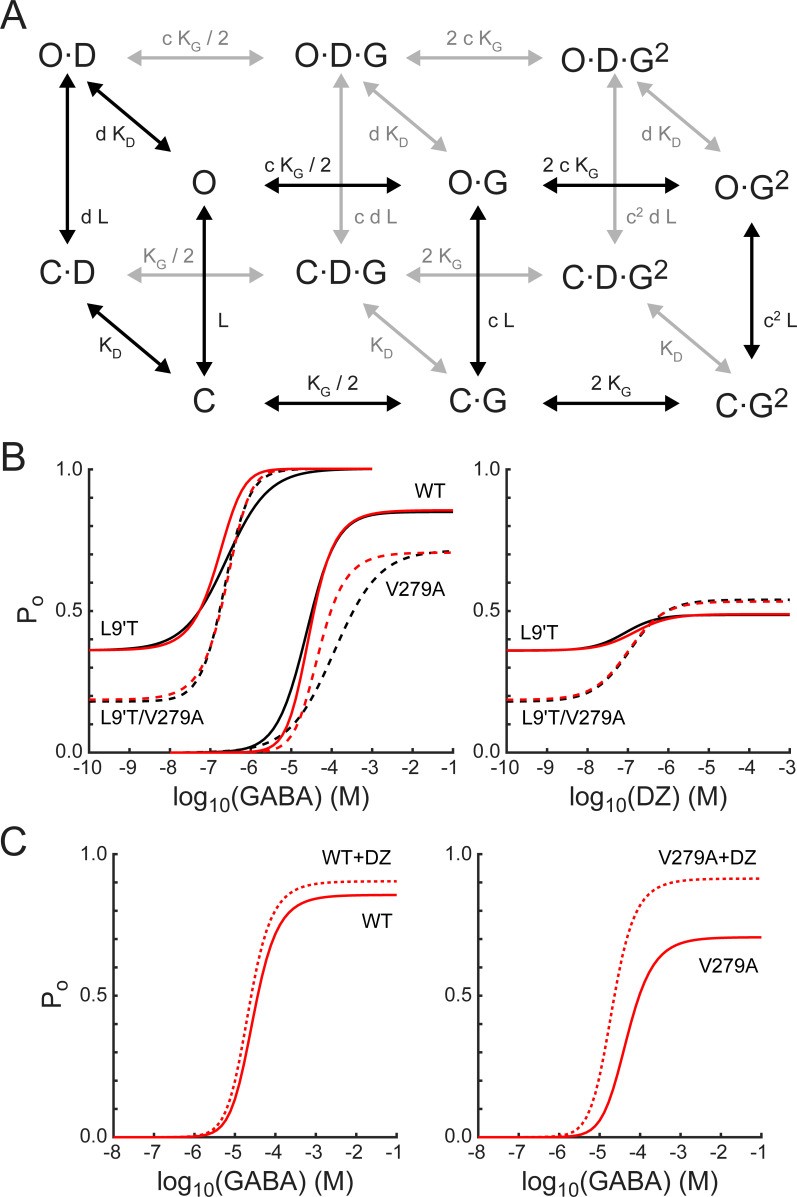
A simple MWC model of channel gating largely accounts for the observed effects of α_1_L9'T and α_1_V279A via independent and additive effects on the pore-gating equilibrium. (**A**) The model depicts channel gating between closed (C) and open (O) states with independent binding of two GABA (G) and one DZ (D) molecule. L is the ratio of closed to open state probabilities in the absence of ligand, K_G_ and K_D_ are the respective dissociation constants for GABA or DZ, and c and d are the respective factors by which GABA or DZ binding influence channel opening. The probability to be in an open state is given by Equation 4. (**B**) Estimated open probability (P_o_) from the data in Figures 2–5 (black) overlaid with model simulations (Equation 4, red). See main text for a detailed description. Model parameters are: L_L9'T_ = 1.8; L_L9'T/V279A_ = 4.4; L_WT_ = 18000; K_G_ = 53 μM; K_D_ = 180 nM; c = 0.0031; d = 0.59; d_V279A_ = 0.20. (**C**) The model’s prediction for potentiation of WT and V279A GABA-evoked responses by 1μM DZ. Figure 9—source code 1.MWC simulation code.

To further explore the idea that α_1_V279A confers its effects primarily by altering pore gating in DZ-bound receptors, we asked whether a simple Monod–Wyman–Changeux (MWC) model of receptor behavior can account for our observations (Figure 9A). This model has been widely used to describe pseudo-steady-state behavior in GABA_A_ receptors (Steinbach and Akk, 2019; Rüsch and Forman, 2005; Chang and Weiss, 1999). For comparison with model simulations, we estimated open probability (P_o_) as a function of GABA or DZ concentration (black curves in Figure 9B) from fits of Equation 1 to the data shown in Figures 2, 4, and 8 where the minimum and maximum P_o_ were scaled according to our observed PTX-sensitive and GABA- or DZ-evoked current amplitudes. In the α_1_L9'T background, the maximum GABA-evoked P_o_ was allowed to vary during optimization, and the minimum unliganded P_o_ was scaled relative to the maximum by the observed mean ratio of I_PTX_/I_GABA-max_ (Figure 3). The maximum DZ-evoked P_o_ was scaled relative to the minimum unliganded P_o_ by the observed mean ratio of I_DZ-max_/I_PTX_ (Figure 5). The maximal P_o_ was set to 0.85 for α_1_β_2_γ_2L_ receptors (Keramidas and Harrison, 2010) and allowed to vary for α_1_V279Aβ_2_γ_2L_ receptors.

**Figure 9—figure supplement 1.**
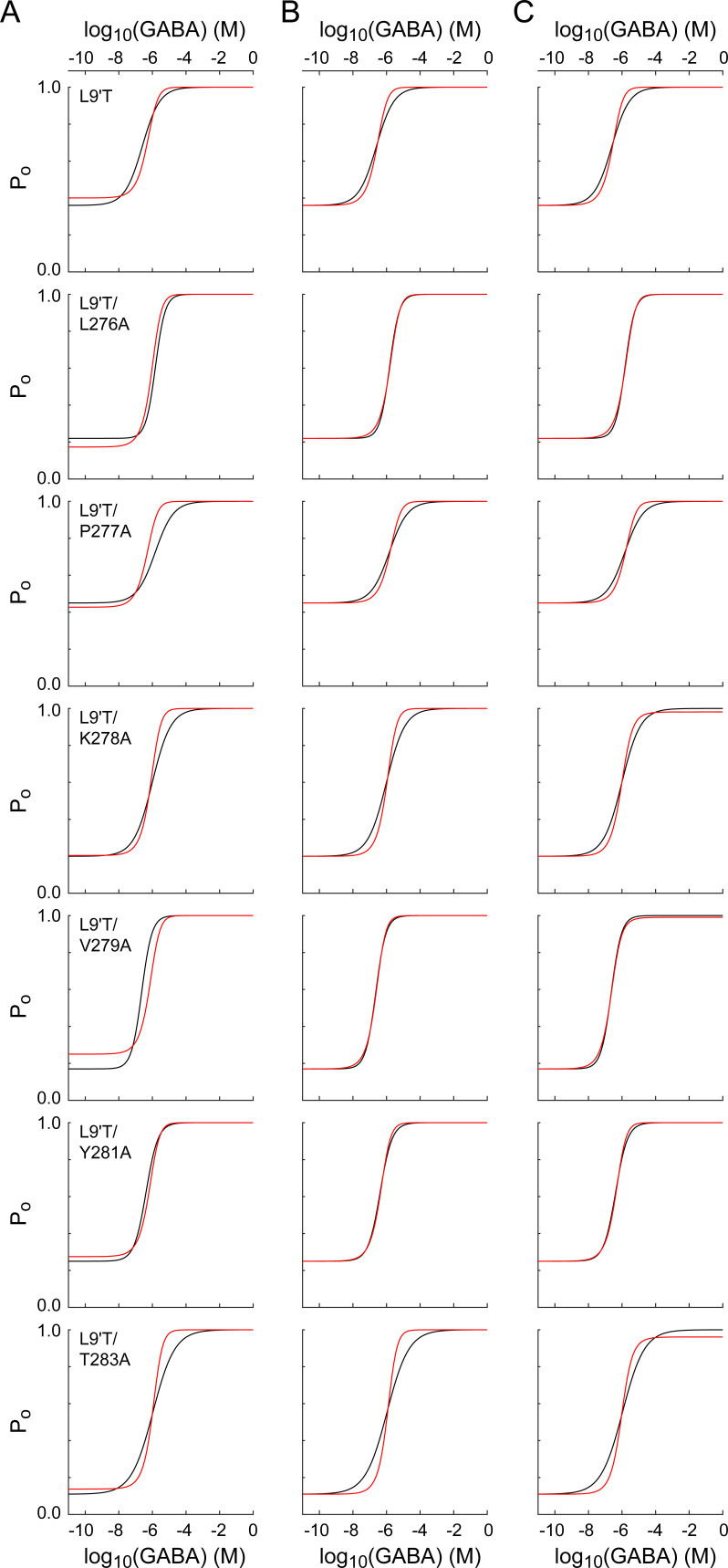
Estimated open probability (P_o_) from the data in Figure 2 (black) overlaid with simulations for the model in Figure 9A (red). (**A**) Model fits assuming mutations have identical affinities for GABA (K_G_ = 190 μM, c constrained to 0.003). L9'T: L = 1.5; L9'T/L276A: L = 0.9; L9'T/P277A: L = 1.3; L9'T/K278A: L = 3.9; L9'T/V279A: L = 0.7; L9'T/Y281A: L = 2.6; L9'T/T283A: L = 6.3. (**B**) Model fits with constraints L =(1 – P_o–unliganded_)/P_o–unliganded_ and c = 0.003. L9'T: K_G_ = 90 μM; L9'T/L276A: K_G_ = 390 μM; L9'T/P277A: K_G_ = 670 μM; L9'T/K278A: K_G_ = 240 μM; L9'T/V279A: K_G_ = 50 μM; L9'T/Y281A: K_G_ = 120 μM; L9'T/T283A: K_G_ = 180 μM. (**C**) Same as (**B**) except c was allowed to vary. L9'T: K_G_ = 270 μM, c = 0.001; L9'T/L276A: K_G_ = 250 μM, c = 0.005; L9'T/P277A: K_G_ = 500 μM, c = 0.004; L9'T/K278A: K_G_ = 8 μM, c = 0.07; L9'T/V279A: K_G_ = 3 μM, c = 0.04; L9'T/Y281A: K_G_ = 270 μM, c = 0.001; L9'T/T283A: K_G_ = 5 μM, c = 0.07.

**Table 1.** Summary of peak current ratios and ΔΔG_DZ_ for mutations in the L9'T background. Data are mean ± standard deviation (# oocytes), and individual data points are shown in Figures 3B, 5B, 6C, and 7C.

	I_PTX_ /I_GABA-max_	I_DZ-max_ /I_PTX_	ΔΔG_DZ_ (kcal/mol)
L9'T	0.36 ± 0.04 (5)	1.35 ± 0.05 (5)	−0.31 ± 0.04 (5)
L9'T/L276A	0.22 ± 0.09 (4)	1.62 ± 0.07 (5)	−0.45 ± 0.04 (5)
L9'T/P277A	0.45 ± 0.03 (3)	1.41 ± 0.17 (5)	−0.46 ± 0.20 (5)
L9'T/K278A	0.20 ± 0.06 (5)	1.67 ± 0.15 (4)	−0.44 ± 0.08 (4)
L9'T/V279A	0.17 ± 0.04 (4)	3.30 ± 0.70 (5)	−1.11 ± 0.30 (5)
L9'T/V279D	0.02 ± 0.005 (3)	2.31 ± 0.08 (3)	−0.52 ± 0.02 (3)
L9'T/V279W	0.16 ± 0.05 (2)	1.96 ± 0.23 (3)	−0.53 ± 0.10 (3)
L9'T/Y281A	0.25 ± 0.07 (5)	1.59 ± 0.11 (4)	−0.42 ± 0.07 (4)
L9'T/T283A	0.11 ± 0.03 (5)	1.71 ± 0.10 (6)	−0.38 ± 0.04 (6)

The model assumes independent GABA and DZ binding. Model parameters were optimized by globally minimizing the sum of squared residuals between simulated (Equation 4) and estimated P_o_ as a function of GABA or DZ concentration for α_1_L9'Tβ_2_γ_2L_, α_1_L9'T/V279Aβ_2_γ_2L_, and α_1_β_2_γ_2L_ receptors (Figure 9B). Responses to α_1_V279Aβ_2_γ_2L_ receptors were not considered during optimization and treated as a model prediction. The mutations α_1_L9'T and α_1_V279A were allowed to perturb the parameter L with the assumption that their effects are independent in the L9'T/V279A double mutant. For wild-type receptors, L was fixed according to its approximate relation with EC_50_ as $L_{WT}=L_{L9T}{\left({EC}_{50,WT}/{EC}_{50,L9T}\right)}^{2}$ (Steinbach and Akk, 2019; Akk et al., 2018; Scheller and Forman, 2002; Chang and Weiss, 1999). The value of d was assumed to be the same for L9'T and wild-type receptors and allowed to vary in the presence of the α_1_V279A mutation. The resulting optimized model parameters (Figure 9 caption) were highly constrained by the data (i.e. estimated P_o_), and the model does a fairly good job of describing our observed responses to either GABA or DZ, differing somewhat in the steepness of their concentration dependence (Figure 9B). Furthermore, the model qualitatively accounts for observations from α_1_V279Aβ_2_γ_2L_ receptors even though they were not considered during optimization. The model’s predicted maximum P_o_ approached 1.0 in the α_1_L9'T background, consistent with a gain of function phenotype. In contrast, maximal P_o_ was reduced in α_1_V279Aβ_2_γ_2L_ as compared to wild-type receptors, as expected for a reduction in unliganded activity. These results suggest that α_1_L9'T and α_1_V279A have independent and additive effects on the gating equilibrium, and that for α_1_V279A, this effect depends on DZ occupancy.

Although responses to GABA or DZ alone are largely explained by this model, DZ potentiation of GABA-evoked responses in a wild-type background is either underestimated or overestimated for subsaturating or saturating GABA concentrations, respectively (Figure 9C). Previous applications of this model to BZD modulation in gain of function backgrounds predict a larger left shift of the P_o_ curve in the presence of drug more similar to that observed, in part due to a smaller value of d (Campo-Soria et al., 2006; Downing et al., 2005; Rüsch and Forman, 2005). However, reducing d also enhances the predicted potentiation of responses to saturating GABA, contrary to that observed for wild-type receptors. Thus, the model is either too simplistic or its assumptions too strict to describe all aspects of DZ potentiation in wild-type channels that both rapidly desensitize and may involve drug modulation of agonist binding and/or intermediate gating steps (Goldschen-Ohm et al., 2014; Gielen et al., 2012). Nonetheless, the model does predict an increase in DZ potentiation of responses evoked by both subsaturating and saturating GABA for α_1_V279A, qualitatively similar to that observed. For responses to saturating GABA in V279A, 42% of the predicted potentiation is accounted for by the lower P_o-GABA-max_ as compared to WT, and the remainder reflects the mutations enhancement of DZ gating.

### Effects of alanine mutations on gating by GABA

To explore the effects of the mutations on gating by GABA, we fit estimated open probability versus GABA concentration relationships using the model in Figure 9A (front face only in the absence of DZ). Estimated open probabilities from fits of Equation 1 to the data in Figure 2 were scaled according to the assumption that P_o-GABA-max _= 1 as described above. We first asked whether all of the mutations could be explained solely by differences in their intrinsic closed–open equilibrium (parameter L). Figure 9—figure supplement 1A shows model predictions where all constructs share identical affinities for GABA (i.e. parameters K_G_ and c). The overall rough qualitative agreement with the data suggests that differences in unliganded open probability are likely to account for much of the observed effects. For comparison, we next constrained L based on the estimated unliganded P_o_ as L = (1 − P_o_)/P_o_ and allowed GABA affinity to differ across constructs. The resulting model fits were slightly improved to a nearly equivalent extent regardless of whether the relative affinity for closed versus open states (parameter c) was held constant (Figure 9—figure supplement 1B) or allowed to vary (Figure 9—figure supplement 1C). Thus, the simplest conclusion is that mutations with right-shifted EC_50_s reduce GABA affinity for the closed state, although we cannot rule out compensatory changes in affinity for closed and open states.

## Discussion

The main conclusions of this study are as follows: First, in the α_1_L9'T gain of function background alanine substitutions in the α_1_M2–M3 linker generally impair unliganded pore opening, indicating that side chain interactions with the linker are important for gating even in the absence of bound agonist. Second, the same mutations have no effect on the amount of chemical energy from DZ binding transmitted to the pore gate, except for α_1_V279A which more than doubles DZ’s energetic contribution to pore gating. Thus, α_1_V279 plays a crucial role to natively hinder drug modulation as compared to its substitution with alanine. Third, introduction of a bulky tryptophan or charged aspartate at position 279 is less favorable than the smaller alanine, suggesting that DZ modulation is inhibited by side chain interactions at the center of the linker. Fourth, α_1_V279D severely impairs unliganded gating. Fifth, α_1_V279A similarly enhances DZ potentiation of GABA-evoked currents in a wild-type background. Sixth, the effects of α_1_V279A in both α_1_L9'T and wild-type backgrounds are explained by specific changes in the pore gating equilibrium and its coupling to DZ binding at the BZD site.

The use of gain of function mutations to study modulatory or weakly activating ligands is well appreciated (Germann et al., 2019; Akk et al., 2018; Campo-Soria et al., 2006; Downing et al., 2005; Rüsch and Forman, 2005; Findlay et al., 2001). Single-channel gating dynamics for combinations of gain of function mutations in AChR suggest that the unliganded and agonist-bound gating mechanisms are similar if not identical and also that they are similarly affected by mutations (Purohit and Auerbach, 2009). Consistent with this idea, MWC models of pseudo steady-state GABA_A_R function have been largely successful in describing the effects of gain of function mutations with changes to the pore gating equilibrium independent of agonist binding (Steinbach and Akk, 2019; Downing et al., 2005; Campo-Soria et al., 2006; Rüsch and Forman, 2005; Chang and Weiss, 1999). The model reported here in Figure 9 supports this conclusion.

Although the effects of α_1_V279A can also be explained by specific changes to the pore-gating equilibrium, they are dependent on DZ occupation of the BZD site. From the model in Figure 9A, we compute an efficiency for GABA of $\eta_{GABA}=[1-log(K_{G})/log(cK_{G})]\times 100\%=37\%$, similar to that reported previously (Nayak et al., 2019). In contrast, DZ efficiency is $\eta_{DZ}=\left[1-log\left(K_{D}\right)/log\left(dK_{D}\right)\right]\times 100\%=3\%$. Whereas α_1_V279A decreases unliganded pore opening, it also increases DZ efficiency ($\eta_{DZ,V279A}=9\%$) to the extent that DZ binding overcomes its intrinsic inhibitory effect and results in similar or even enhanced activation as compared to wild type (Figure 9). Interestingly, an M2–M3 linker mutation in the γ_2_ subunit was similarly observed to impair activation by GABA and enhance modulation by the general anesthetic propofol (O'Shea et al., 2009).

Our estimates for the energetic effect of DZ binding on pore gating (−0.4 kcal/mol) are similar to prior estimates from direct gating in L9'S/T backgrounds and from kinetic models (Goldschen-Ohm et al., 2014; Gielen et al., 2012; Downing et al., 2005; Rüsch and Forman, 2005). This is approximately 10-fold less than the energy derived from binding each molecule of GABA (Goldschen-Ohm et al., 2014; Maksay, 1994; Jackson, 1992). However, it is sufficient to produce a relevant change in open probability in channels with small free energy differences between closed and open states, such as conferred by single bound agonists, partial agonists, other allosteric modulators, or gain of function mutations. From a clinical perspective, such small perturbations are likely to be more easily tolerated in patients. Given that the M2–M3 linker is associated with early movements during AChR activation (Purohit et al., 2013), it is possible that biasing the linker toward its active conformation in the α_1_L9'T background could limit our ability to observe its full range of motion. In this case, our observed effects of linker mutations on DZ modulation may underestimate their effects on the full activation pathway.

The interface between the extracellular agonist- and BZD-binding domains and the transmembrane helices is formed largely by several loops including the M2–M3 linker, Cys loop, β1–β2 loop and β8–β9 linker, and pre-M1 segment from neighboring subunits (Figure 1). Mutagenesis suggests that interactions between these domains are important for channel gating by agonists (Kash et al., 2003; O'Shea and Harrison, 2000) thought to involve an outward radial displacement of the M2–M3 linker (Nemecz et al., 2016). Rate versus free energy linear relationships in AChR suggest that the M2–M3 linker moves early during channel activation, similar to rearrangements at agonist binding sites (Purohit et al., 2013). Consistent with these observations, mutant cycle analysis indicates strong long-range coupling between residues in the M2–M3 linker and agonist binding sites (Gupta et al., 2017). Given the homology between the BZD site at the α/γ interface and agonist sites at β/α interfaces, interactions between the M2–M3 linker and BZD site are reasonable, although they need not be very strong given the relatively small energy DZ contributes to pore gating. As with agonist sites, such interactions could be mediated by global backbone conformational fluctuations or via distinct structural components, or both. Either way, side chain interactions at position 279 in the α_1_ subunit play an important role in coupling between the BZD site and pore gate.

In structural models, the M2–M3 linker adopts a C-shaped conformation with α_1_V279 oriented inwards toward the center of the arc (Figure 1D; Kim et al., 2020; Laverty et al., 2019; Masiulis et al., 2019; Phulera et al., 2018; Zhu et al., 2018). We hypothesize that the side chain at position 279 is centrally involved in steric interactions between linker residues near the top of the M2 and M3 helices. Removing this obstruction (α_1_V279A) would allow the linker to become more compact and bring the M2 and M3 helices closer together. In contrast, similar or larger side chains (α_1_V279D/W) would sterically inhibit such a conformational change. In this case, we speculate that the closer proximity of the M3 helix could impair unliganded pore gating by hindering radial expansion of the pore lining M2 helix. Conversely, a more compact transmembrane domain may also enhance coupling with the BZD site (Kim et al., 2020). Valine and threonine residues in GlyR and AchR aligning with V279 (Figure 1—figure supplement 1) adopt a similar conformation (Kumar et al., 2020; Nemecz et al., 2016; Du et al., 2015), suggesting that this residue has a conserved role in other pLGICs.

Alternatively, removing most of the central side chain may simply alter linker flexibility. Linker flexibility has been suggested to be inversely correlated with gating, where stabilizing interactions between α_1_R19' (located just below α_1_V279) and the linker backbone may promote coupling between extracellular and intracellular domains (Masiulis et al., 2019). Thus, the strong inhibition of unliganded opening conferred by α_1_V279D could reflect competition for α_1_R19', thereby weakening its interaction with the linker backbone and increasing linker flexibility. However, this does not provide a simple explanation for the opposing effects of α_1_V279A on unliganded versus DZ-bound gating. Regardless, it is important to keep in mind that the energetic changes that we observe for DZ gating are on the order of a hydrogen bond or two, and thus can be accounted for by relatively subtle changes.

The β8-β9 linker and pre-M1 in the neighboring γ_2_ subunit come in close proximity to the α_1_M2–M3 linker and are known to be important for BZD modulation, with the β8-β9 linker also contributing to the BZD-binding site (Hanson and Czajkowski, 2011; Hanson and Czajkowski, 2008). In AChR strong coupling with the adjacent β8-β9 linker and pre-M1 segment of the neighboring subunit occurs primarily via the threonine aligning with α_1_V279 in GABA_A_R and its neighboring serine (Gupta et al., 2017). A reorientation of the linker due to removal of steric interactions near its center could alter coupling to the BZD site via interactions with the γ_2_ β8-β9 linker and/or pre-M1. For example, a compression of the ends of the M2–M3 linker would cause the middle of the linker to be squeezed outward toward the neighboring γ_2_ subunit, potentially enhancing intersubunit coupling. Comparison of GlyR structures in apo or antagonist-bound versus agonist-bound conformations indicates that intersubunit interactions between the M2–M3 linker in the vicinity of the valine aligning with V279 and the following serine, and the top of the M1 helix in the adjacent subunit are weakened during channel activation (Du et al., 2015; Kumar et al., 2020). Thus, a stronger intersubunit coupling in this area could potentially both reduce unliganded opening and enhance coupling with rearrangements of the γ_2_ subunit upon BZD binding. However, other regions including the β4-β5 linker in the channel’s outer vestibule also affect BZD modulation of agonist-evoked currents (Pflanz et al., 2018; Venkatachalan and Czajkowski, 2012). Thus, BZD modulation likely involves larger domain fluctuations in addition to any specific molecular pathways involving α_1_V279.

Recent structures of αβγ receptors show DZ bound not only at the classical α/γ site in the extracellular domain, but also in the transmembrane domain below the M2–M3 linker at β/α and γ/β intersubunit interfaces (Kim et al., 2020; Masiulis et al., 2019). Thus, it is intriguing to speculate that enhanced DZ gating as conferred by α_1_V279A may involve occupation of a transmembrane site. However, these structures were solved in the presence of 100–200 μM DZ, and the relevance of these transmembrane sites to high affinity binding of ~1 μM DZ at the classical site is unclear (Walters et al., 2000). Also, DZ binding in the transmembrane domain was not observed at α/γ or α/β interfaces. Therefore, we favor the interpretation that our observed effects reflect altered functional coupling with the classical site.

Our observations reveal a critical residue V279 in the α_1_M2–M3 linker, which regulates energetic coupling between the BZD site and the pore gate, whereas other linker side chains contribute little or not at all. These data shed new light on the molecular basis for GABA_A_R modulation by one of the most widely prescribed classes of psychotropic drugs.

## Materials and methods

### Mutagenesis and expression in oocytes

DNA for wild-type GABA_A_R rat α_1_, β_2_, and γ_2L_ subunits were a gift from Dr. Cynthia Czajkowski. Single alanine substitutions were introduced throughout the α_1_M2–M3 linker from L276-T283 in addition to the gain of function α_1_L9'T pore mutation (rat α_1_L263T) (QuikChange II, Qiagen). Mutations V279D and V279W were introduced similarly. Each construct was verified by forward and reverse sequencing of the entire gene. mRNA for each construct was generated (mMessage mMachine T7, Ambion) for expression in *X. laevis* oocytes (EcoCyte Bioscience, Austin, TX). Oocytes were injected with 27–54 ng of total mRNA for α, β, and γ subunits in a 1:1:10 ratio (Boileau et al., 2002) (Nanoject, Drummond Scientific). Oocytes were incubated in ND96 (in mM: 96 NaCl, 2 KCl, 1 MgCl_2_, 1.8 CaCl_2_, 5 HEPES, pH 7.2) with 100 mg/ml gentamicin at 18°C.

### Two-electrode voltage clamp recording and analysis

Currents from expressed channels 1–3 days post-injection were recorded in two-electrode voltage clamp (Dagan TEV-200, HEKA ITC and Patchmaster software). Oocytes were held at −80 mV and perfused continuously with buffer (ND96) or buffer containing PTX, GABA, or DZ. PTX was diluted from a 1 M stock solution in DMSO. DZ was diluted from a 10 mM stock solution in DMSO. Fresh PTX and DZ stock solutions were tested several times with no change in results. GABA was dissolved directly from powder. A microfluidic pump (Elveflow OB1 MK3+) and rotary valve (Elveflow MUX Distributor) provided consistent and repeatable perfusion and solution exchange across experiments, which limited solution exchange variability to primarily differences between oocytes only. Ten second pulses of PTX, GABA, or DZ were followed by 3–6 min in buffer to allow currents to return to baseline. Recorded currents were analyzed with custom scripts in MATLAB (Mathworks). Recordings of concentration–response relationships were bookended by pulses of PTX to correct for any drift or rundown during the experiment. Briefly, currents were baseline subtracted to correct for drift and then scaled by a linear fit to the peak of each PTX response to correct for rundown (Figure 2—figure supplement 1). The amount of current rundown was variable across oocytes, with no clear relation to specific constructs. Concentration–response relationships were fit to the Hill equation:

(1) $$\frac{I}{I_{max}}=\frac{1}{1+{\left(\frac{{EC}_{50}}{\left[X\right]}\right)}^{n}}$$

 where I is the peak current response, $\left[X\right]$ is ligand concentration, ${EC}_{50}$ is the concentration eliciting a half-maximal response, and n is the Hill slope.

### Single-channel recording

HEK293T cells were transfected with DNA for rat α_1_L9'T/V279A, β_2_ and γ_2L_ subunits in a 1:1:1 ratio. Single-channel currents were recorded 16–32 hr post-transfection from excised inside-out patches clamped at −80 mV. Currents were acquired at 20 kHz and low-pass filtered at 2 kHz (Axopatch 200A, HEKA ITC, and PatchMaster software). Extracellular (pipet) solution was (in mM): 145 NaCl, 2.5 KCl, 2 CaCl_2_, 1 MgCl_2_, 10 HEPES, 4 Dextrose, pH 7.3 with NaOH. Intracellular (bath) solution was (in mM): 140 KCl, 10 EGTA, 10 HEPES, 2 MgATP, pH 7.3 with KOH. GABA was dissolved in the extracellular solution.

### DZ-gating model

For the model in Figure 7A, the free energy difference for unliganded (ΔG_0_) and DZ-bound (ΔG_1_) gating was estimated as follows:

(2) $$\Delta G_{0}=-kT\;ln{\left(\frac{P_{open}}{P_{closed}}\right)}_{unliganded}\approx -kT\;ln\left(\frac{P_{o-GABA-max}\left(I_{PTX}/I_{GABA-max}\right)}{1-P_{o-GABA-max}\left(I_{PTX}/I_{GABA-max}\right)}\right)$$

(3) $$\Delta G_{1}=-kT\;ln{\left(\frac{P_{open}}{P_{closed}}\right)}_{DZ-bound}\approx -kT\;ln\left(\frac{P_{o-GABA-max}\left(I_{DZ-max}/I_{GABA-max}\right)}{1-P_{o-GABA-max}\left(I_{DZ-max}/I_{GABA-max}\right)}\right)$$

where $I_{PTX}$, $I_{GABA-max}$, and $I_{DZ-max}$ are as shown in Figures 3 and 5, *k* is the Boltzmann constant, *T* is temperature, and $P_{o-GABA-max}$ is the open probability in saturating GABA, which was assumed to be approximately 1. Importantly, even if this assumption is incorrect, the effect on DZ’s energetic contribution to pore gating should be minimal and our overall conclusion unchanged (see Figure 7B). Also, this assumption was verified for single L9'T/V279A receptors (Figure 7—figure supplement 1).

### MWC model

For the model in Figure 9A, the probability to be in an open (O) state is given by:

(4) $$P_{o}={\left(1+L\frac{\left(1+\frac{\left[DZ\right]}{K_{D}}\right){\left(1+\frac{\left[GABA\right]}{K_{G}}\right)}^{2}}{\left(1+\frac{\left[DZ\right]}{dK_{D}}\right){\left(1+\frac{\left[GABA\right]}{cK_{G}}\right)}^{2}}\right)}^{-1}$$

where L is the ratio of closed (C) to open (O) state probabilities in the absence of ligand, K_G_ and K_D_ are the respective dissociation constants for GABA or DZ, and c and d are the respective factors by which GABA or DZ binding influence pore opening.

## Data Availability

Source code for the modeling depicted in Figure 9 has been provided.

## Additional files

Transparent reporting form
